# Ongoing HIV Transmission and the HIV Care Continuum in North Carolina

**DOI:** 10.1371/journal.pone.0127950

**Published:** 2015-06-04

**Authors:** Anna B. Cope, Kimberly A. Powers, JoAnn D. Kuruc, Peter A. Leone, Jeffrey A. Anderson, Li-Hua Ping, Laura P. Kincer, Ronald Swanstrom, Victoria L. Mobley, Evelyn Foust, Cynthia L. Gay, Joseph J. Eron, Myron S. Cohen, William C. Miller

**Affiliations:** 1 Department of Epidemiology, University of North Carolina at Chapel Hill, Chapel Hill, North Carolina, United States of America; 2 Division of Infectious Diseases, Department of Medicine, School of Medicine, University of North Carolina at Chapel Hill, Chapel Hill, North Carolina, United States of America; 3 Bristol-Myers Squibb, Lawrenceville, New Jersey, United States of America; 4 Lineberger Comprehensive Cancer Center, University of North Carolina at Chapel Hill, School of Medicine, Chapel Hill, North Carolina, United States of America; 5 Department of Biochemistry and Biophysics, University of North Carolina at Chapel Hill, Chapel Hill, North Carolina, United States of America; 6 UNC Center for AIDS Research, University of North Carolina at Chapel Hill, School of Medicine, Chapel Hill, North Carolina, United States of America; 7 North Carolina Department of Health and Human Services, Division of Public Health, Raleigh, North Carolina, United States of America; University of Alabama at Birmingham, UNITED STATES

## Abstract

**Objective:**

HIV transmission is influenced by status awareness and receipt of care and treatment. We analyzed these attributes of named partners of persons with acute HIV infection (index AHI cases) to characterize the transmission landscape in North Carolina (NC).

**Design:**

Secondary analysis of programmatic data.

**Methods:**

We used data from the NC Screening and Tracing of Active Transmission Program (2002–2013) to determine HIV status (uninfected, AHI, or chronic HIV infection [CHI]), diagnosis status (new or previously-diagnosed), and care and treatment status (not in care, in care and not on treatment, in care and on treatment) of index AHI cases' named partners. We developed an algorithm identifying the most likely transmission source among known HIV-infected partners to estimate the proportion of transmissions arising from contact with persons at different HIV continuum stages. We conducted a complementary analysis among a subset of index AHI cases and partners with phylogenetically-linked viruses.

**Results:**

Overall, 358 index AHI cases named 932 partners, of which 218 were found to be HIV-infected (162 (74.3%) previously-diagnosed, 11 (5.0%) new AHI, 45 (20.6%) new CHI). Most transmission events appeared attributable to previously-diagnosed partners (77.4%, 95% confidence interval 69.4–85.3%). Among these previously-diagnosed partners, 23.2% (14.0–32.3%) were reported as in care and on treatment near the index AHI case diagnosis date. In the subset study of 33 phylogenetically-linked cases and partners, 60.6% of partners were previously diagnosed (43.9–77.3%).

**Conclusions:**

A substantial proportion of HIV transmission in this setting appears attributable to contact with previously-diagnosed partners, reinforcing the need for improved engagement in care after diagnosis.

## Introduction

Antiretroviral treatment (ART) reduces the probability of HIV transmission by suppressing plasma viral load (VL) to undetectable levels [1–3]. However, current estimates of the HIV care continuum, or cascade, indicate that most HIV-infected persons in the US are not achieving viral suppression [4, 5]. Undiagnosed persons and those diagnosed but not in care, on treatment and virally suppressed are potential sources of ongoing transmission and high-priority targets for maximizing HIV prevention.

Current estimates of the transmission contributions made by persons aware and unaware of their HIV status are based on mathematical models [6–9]. Empirical estimates to compare to modeling studies are difficult to obtain, as they require information at the time of HIV acquisition about newly infected persons’ transmitting partners. However, few people are diagnosed near the time of HIV transmission, [10–15] and many do not know with certainty from whom they acquired HIV.

The North Carolina (NC) Screening and Tracing of Active Transmission (STAT) program has detected persons with acute HIV infection (AHI) since 2002, providing a unique opportunity to characterize the partners of newly infected persons near the time of transmission [10, 16]. The primary objective of this secondary analysis of STAT data is to classify the HIV status and diagnosis, care, treatment, and viral suppression status of a) all traceable partners and b) the most likely transmission source among identified HIV-infected partners for acutely-infected persons (index AHI cases) diagnosed in NC between 2002 and 2013. The first analysis characterizes the overall continuum-related landscape in a network where HIV incidence is known to be actively occurring; the second describes the continuum attributes of persons specifically deemed the most likely transmission source for incident cases. The overarching goal of this analysis is to provide information for designing, modeling, and targeting HIV care and treatment services.

## Methods

For each index AHI case aged ≥16 years and diagnosed between November 2002 and June 2013 in NC, we assessed the HIV status and diagnosis, care, and treatment status (if available) of sexual and needle-sharing partners he/she named at the time of diagnosis. Data for the main analyses originated from the STAT program, a NC Division of Public Health (DPH) effort to identify persons with AHI. In a complementary analysis, we used data from the Center for HIV/AIDS Vaccine Immunology 001 Study: Acute HIV Infection Prospective Cohort Study (CHAVI-001), an observational study examining factors related to HIV transmission [17].

### STAT Program

The STAT program identifies index AHI cases through testing at publicly-funded sites (e.g., health departments) and (since 2006) community based organizations/settings (e.g. private providers) [10, 16]. AHI is defined as: a) a negative or indeterminate antibody test and reproducibly detectable HIV RNA, or b) a positive antibody test with seronegative documentation within 30 days. The number of publicly-funded sites increased from 110 in 2002 to 149 in 2012 [10, 18]. Report of AHI in community settings is dependent upon recognition of testing results indicative of AHI by either the individual providers and/or state health officials.

Disease intervention specialists (DIS) contact index AHI cases within 72 hours of release of HIV test results and perform an initial interview, conduct confirmatory HIV testing, and make referrals to HIV care providers. DIS also attempt to find, counsel, and provide HIV testing for all named partners during the 8 weeks prior to the index AHI case’s diagnosis date.

#### Partner HIV Status Determination

All partners were classified as identifiable or anonymous. Anonymous partners were not pursued by DIS because identifying information provided by the index AHI case was absent or incomplete. For identifiable partners, the index AHI case provided enough information for DIS to identify the partner and classify him/her as “previously-diagnosed” or “undiagnosed” based on a search of electronic NC HIV surveillance databases (if the partner resided in NC) or consultation with health departments outside of the state (if the partner did not reside in NC). Undiagnosed partners who were not located or not willing to be tested were classified as “unknown.” The remaining undiagnosed partners were tested for HIV and classified as “HIV-uninfected,” “new AHI,” or “new chronic HIV infection (CHI).” For all HIV-infected partners not diagnosed with AHI or AIDS within 6 months of the index AHI case diagnosis, results of the Serologic Testing Algorithm for Recent HIV Seroconversion (STARHS) were used (when available) to approximate recent (≤6 months) and longstanding (>6 months) infection at the time of transmission, based on a normalized optical density cut-point of <0.8 on the BED assay [19]. All partners diagnosed >6 months before the index AHI case and those diagnosed ≤6 months before but received an AIDS diagnosis within 6 months of the index AHI case diagnosis were classified as longstanding infection.

#### Partner Diagnosis, Care, Treatment, and Viral Suppression Status

Per STAT program protocol, DIS investigate care-seeking and treatment behaviors of previously-diagnosed partners in the 6 months before the index AHI case’s diagnosis for classification as “in care” (HIV provider-confirmed visit and/or 1 clinical lab present in surveillance databases) and/or “on ART” (HIV provider-confirmed receipt of ART and/or a VL below the detectable limits of the reported test in surveillance databases).

We classified the diagnosis status (new AHI, new CHI, or previously-diagnosed) of all HIV-infected partners and the reported care and treatment status at the time of the index AHI case diagnosis (not in care, in care and not on ART, in care and on ART, unclassified care and treatment) of all previously-diagnosed partners based on DIS report. Because quantitative VLs are not collected as part of the STAT program, we extracted all partner VLs reported in the NC electronic HIV/AIDS reporting system (eHARS) between 6 months before to 2 months after the index AHI case diagnosis date. To assess viral suppression (<200 copies/ml) status near transmission, we considered only the closest VL to the index AHI case’s diagnosis date. For previously-diagnosed partners with VLs before and after the index AHI case diagnosis date, the closest VL before diagnosis was used to assess viral suppression near transmission.

#### Likely Transmission Source Identification

Each index AHI case was classified according to the HIV status pattern of named partners (first four columns of Table 1): A) the HIV status of all partners was known and only one was HIV-infected, B) >1 HIV-infected partner (with or without additional unknown/anonymous partners) or one HIV-infected partner with additional unknown/anonymous partners, C) only partners of unknown/anonymous status, and D) no HIV-infected or unknown/anonymous partners.

**Table 1:** Index AHI cases by pattern of partner HIV status.

Type	Number of known HIV-infected partners	Number of known HIV-uninfected partners	Number of unknown status partners	Number of index AHI cases with partner HIV status pattern	
				N	%
**A**	1	≥0	0	**106**	**(29.6)**
**B**				**68**	**(19.0)**
* B1*	*>1*	*≥0*	*0*	*12*	*(3*.*4)*
* B2*	*1*	*≥0*	*≥1*	*38*	*(10*.*6)*
* B3*	*>1*	*≥0*	*≥1*	*18*	*(5*.*0)*
**C**	0	0	≥1	**127**	**(35.5)**
**D**				**57**	**(15.9)**
* D1*	*0*	*0*	*0*	*38*	(10.6)
* D2*	*0*	*≥1*	*0*	*19*	(5.3)

For index cases naming only 1 HIV-infected partner with the status of all other partners known (Type A), this single HIV-infected partner was assumed to be the most likely transmitting partner. For index AHI cases naming >1 potential transmitting partner (i.e., known HIV-infected or status-unknown partner) with at least one confirmed HIV-infected partner (Type B), we assumed that the likely transmitting partner was among those partners named. We repeatedly, randomly sampled the known HIV-infected partners 1000 times with replacement to identify the most likely transmitting partner. For index AHI cases naming 1 HIV-infected partner (Type B2), the probability of selecting that partner was 1, while the probability of selection for each partner of index AHI cases naming >1 HIV-infected partner (Types B1, B3) was 1/(number of named HIV-infected partners). Index AHI cases not naming any HIV-infected partner (Types C and D) were excluded from these analyses. For these cases, the diagnosis, care and treatment status for the most likely transmission source among identified partners could not be estimated because all partners were HIV-uninfected or of unknown status.

#### HIV Care Continuum Analyses

To describe the overall transmission landscape in this network, we calculated the proportion of all HIV-infected partners with each diagnosis, care, and treatment status. To describe the putative transmission contributions of the various continuum stages, we calculated these proportions only among the persons identified as the most likely transmission source for each incident case. Although no single sample from our repeated sampling approach captures all transmitting partners, the combination of the samples provides a reasonable estimate of the range of plausible values for the diagnosis, care, and treatment status of the most-likely transmitting partner among named HIV-infected partners [20].

### CHAVI-001

Between 2006 and 2011, all index AHI cases identified via the STAT program were referred for evaluation at the University of North Carolina at Chapel Hill (UNC-CH) or Duke University, and offered enrollment in CHAVI-001. Study investigators collected basic demographics for each enrolled index AHI case (representing a subset of the STAT index AHI cohort) and data for all partners within 12 weeks of their diagnosis date. Locatable partners were offered enrollment in CHAVI-001, regardless of HIV status.

Upon enrollment, cell-free plasma was extracted from samples provided by index AHI cases and HIV-infected partners to isolate viral RNA using the QIAMP Viral RNA Mini Kit (Qiagen). DNA sequence alignments, phylogenetic tree generation, and transmission pair confirmation were performed on either full length *env* genes resulting from single genome amplification or *pro-pol* amplicons derived from bulk sequencing as previously described [21–30]. The donor was distinguished from the recipient among all phylogenetically-linked transmission pairs, using Bayesian Evolutionary Analysis by Sampling Trees (BEAST) v.1.4.8, as previously described [21, 31]. All phylogenetic sequences were or will be submitted to GenBank. Accession numbers for selected sequences include: EU578952-EU578997, HQ908232-HQ908254, HQ908150-HQ908183, HQ908139-HQ908149, and HQ908184-HQ908218. The remaining GenBank accession numbers will be provided upon acceptance.

#### CHAVI-001 Data Analysis

The HIV status of partners named by index AHI cases enrolled in CHAVI-001 was assigned based on CHAVI-001 field investigation and the proportion of phylogenetically-linked partners with each diagnosis status was estimated. We based treatment status of phylogenetically-linked partners on reported ART history collected at enrollment. Partner care status was not collected for study purposes and therefore not assigned. Data from the STAT program could not be linked to CHAVI-001 partners for reasons of confidentiality.

All statistical analyses were conducted in SAS version 9.3 (Cary, NC).

### Ethics Statement

The University of North Carolina at Chapel Hill Biomedical Institutional Review Board approved this study. All data collected as part of the STAT program was collected for surveillance purposes for the NC DPH and consent was not required from index AHI cases at diagnosis, regardless of age. However, prior to 2012, index AHI cases identified in NC did sign a Health Insurance Portability and Accountability Act (HIPAA) form to allow DIS to report detailed demographic and testing information about named partners for the STAT program. In 2013, the HIPAA form was no longer deemed necessary by the NC DPH because the STAT program was considered a state-sponsored surveillance program. The NC DPH and the University of North Carolina at Chapel Hill Biomedical Institutional Review Board approved this consent procedure for the STAT program. All patients who enrolled on CHAVI-001 provided written informed consent.

## Results

### STAT

#### Index AHI cases

Between November 2002 and June 2013, 358 index AHI cases were identified via the STAT program. Index AHI cases were predominantly Black (70.1%), male (83.0%) and self-identified as men who have sex with men (MSM) (66.5%). Nearly one-quarter of cases had been diagnosed with a sexually transmitted infection within 2 month of their AHI diagnosis (24.3%) [Table 2]. The majority of index AHI cases were diagnosed through the state laboratory at publicly-funded sites (71.2%). A higher proportion of index AHI cases diagnosed at publicly-funded testing sites had been diagnosed with an STI in the 8 weeks prior to their HIV diagnosis as compared to those diagnosed in community settings (27.5% versus 16.5%). Cases diagnosed at publicly-funded testing sites were similar to those diagnosed in community settings across all other demographic and risk categories.

**Table 2:** Demographics of Index AHI and Named Partners.

	STAT Program*				CHAVI-001**			
	Index AHI		Named Partners^		Index AHI		Named Partners^@^	
	(N = 358)		(N = 656)		(N = 117)		(N = 367)	
	Median	(IQR)	Median	(IQR)	Median	(IQR)	Median	(IQR)
**Age at Diagnosis**	26	(21–36)	28	(23–37)	25	(21–36)	27	(22–35)
**Number of Named Partners**	2	(1–3)	--	--	3	(2–4)	--	--
	**N**	**(%)**	**N**	**(%)**	**N**	**(%)**	**N**	**(%)**
**Reporting Location**								
State Laboratory (NAAT Pooling)	255	(71.2)	--	--	--	--	--	--
Community Setting	103	(28.8)	--	--	--	--	--	--
**Gender**								
Female	61	(17.0)	85	(13.0)	15	(12.8)	40	(10.9)
Male	297	(83.0)	563	(85.8)	102	(87.2)	327	(89.1)
**Sex Risk**								
Female	61	(17.0)	85	(13.0)	15	(12.8)	40	(10.9)
Heterosexual Male	39	(10.9)	95	(14.5)	13	(11.1)	26	(7.1)
MSM	238	(66.5)	468	(71.3)	89	(76.1)	300	(81.7)
Unknown Risk Male	20	(5.6)	--	--	--	--	--	--
**Race** ^**#**^								
Black	251	(70.1)	411	(62.7)	76	(65.0)	237	(64.6)
White, Non-Hispanic	82	(22.9)	160	(24.4)	35	(29.9)	106	(28.9)
White, Hispanic	20	(5.6)	43	(6.6)	6	(5.1)	8	(2.2)
Other	3	(0.8)	2	(0.3)	--	--	--	--
**STI in 8 weeks prior to AHI Diagnosis**								
Yes	87	(24.3)	--	--	20	(17.1)	--	--
No	271	(75.7)	--	--	97	(82.9)	--	--

*The North Carolina Screening and Tracing of Active Transmission Program. The STAT cases are inclusive of CHAVI-001 cases.

** Center for HIV/AIDS Vaccine Immunology 001 Study: Acute HIV Infection Prospective Cohort Study.

^^^ 932 partners were named by Index AHI cases during the STAT investigation. Index AHI cases refused to provide detailed demographic information for the STAT Program for a total of 276 partners, leaving 656 with detailed demographic information.

^@^ One partner identified in CHAVI-001 was missing gender.

^#^ Missing Race not included in table.

#### Partner Overview

**Fig 1:**
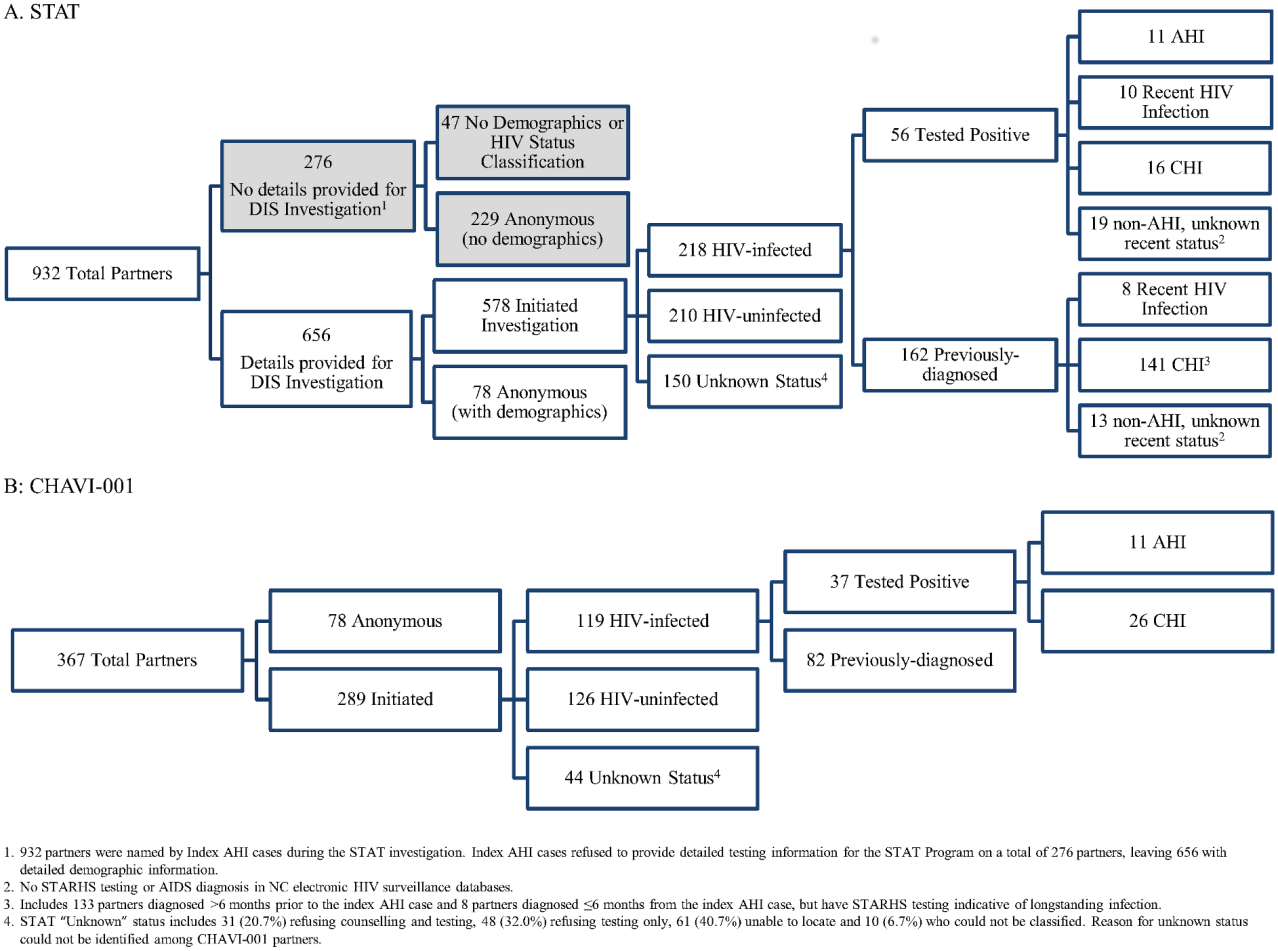
HIV Status of sexual and needle-sharing partners reported by index AHI cases. (A) HIV status of partners of index AHI cases identified via the STAT program between November 2002 and June 2013. (B) HIV status of partners reported by index AHI cases enrolled in CHAVI-001 between May 2006 and June 2011.

Overall, 932 sexual partners (4 of whom were also needle-sharing) were reported by index AHI cases in the 8 weeks prior to their diagnosis (per-index median = 2; range 0–27). Index AHI cases provided detailed information for 656 partners (70.4%) as part of the STAT program [Fig 1a]. Most partners with detailed information were male (85.8%) and Black (62.7%), with a median age of 28 years (IQR 23–37) [Table 2].

Of the 656 partners with detailed information, 218 (33.2%) were HIV-infected. Of these, 162 (74.3%) were previously-diagnosed and 56 (25.7%) were newly-diagnosed (11 AHI and 45 CHI). An additional 210 partners (32.0%) were HIV-uninfected (antibody and HIV RNA negative) at DIS follow-up. The HIV status for 228 partners (34.8%) remained undetermined after the DIS investigation (78 anonymous, 31 counseling-and-testing refusals, 48 testing-only refusals, 71 unlocatable or unclassified based on DIS reports) [Fig 1a].

Recent HIV infection could be assessed for 186 (85.3%) HIV-infected partners not diagnosed during AHI. Of the 162 previously-diagnosed partners, 133 (82.1%) were diagnosed >6 months before the index AHI case’s diagnosis date and therefore classified as longstanding infection. Among the remaining 29 previously-diagnosed partners with the possibility of recent infection, only 16 (55.2%) had STARHS testing results, of whom 8 (50.0%) were considered recently infected. Of the 45 newly-diagnosed partners not diagnosed during AHI, 26 (57.8%) had STARHS testing available, of whom 10 (38.5%) were considered recently infected. A total of 5 (62.5%) of the 8 previously-diagnosed, recently-infected partners had a VL reported in the NC eHARS between 6 months before to 2 months after the index AHI case’s diagnosis date; all 5 were unsuppressed. Of these unsuppressed partners, DIS reported 2 to not be in care prior to the index AHI diagnosis date, 2 were in care but not on ART, and 1 was in care and on ART. Including new AHI diagnoses, a total of 29 (13.3%) HIV-infected partners were estimated to have been infected within 1 year of the index AHI case.

**Fig 2:**
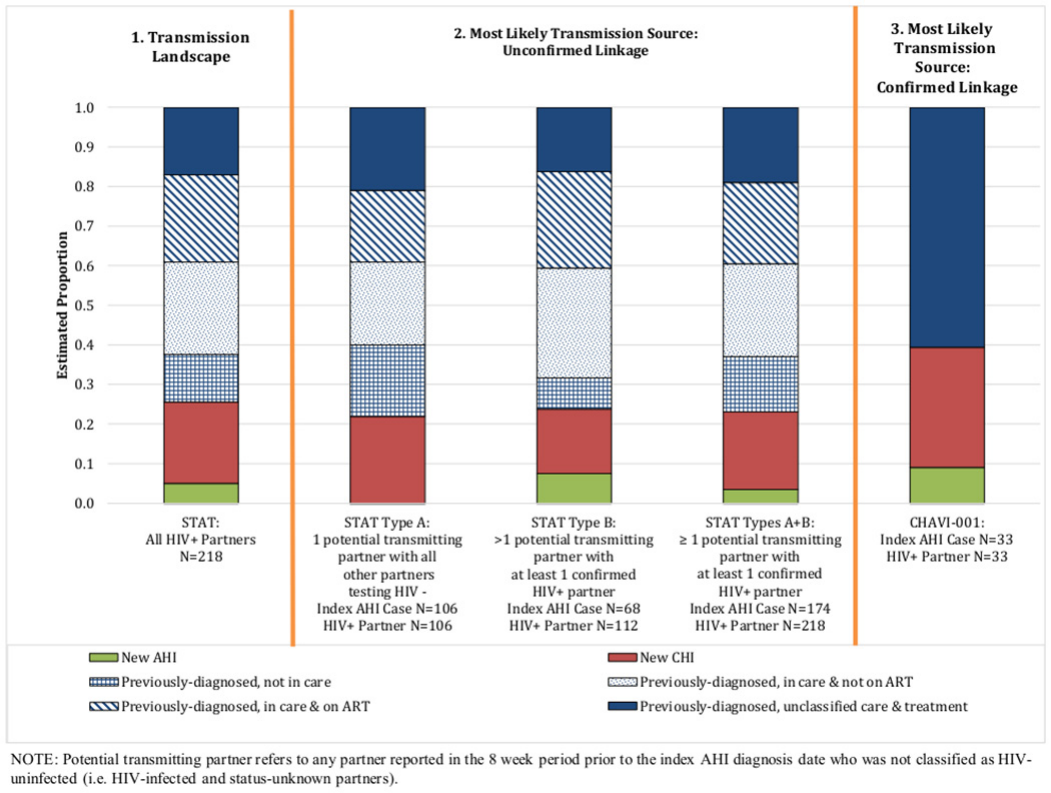
HIV care continuum stages for HIV-infected partners. The diagnosis status and the care and treatment status (if available) are presented for 1) all HIV-infected partners named by the index AHI cohort and identified by the STAT program, 2) the most likely transmission source among identified HIV-infected partners presented by the pattern of HIV-infected, uninfected, and status-unknown partners reported to the STAT program (unconfirmed linkage) and 3) phlyogenetically-linked partners identified via the CHAVI-001 study (confirmed linkage). For the data originating from the STAT program, repeated random sampling was used to estimate diagnosis, care, and treatment status of the most likely transmission source when >1 potential transmitting partner was named.

Among all 162 previously-diagnosed partners (most of whom were chronically infected), 26 (16.0%) were not in care, 51 (31.5%) were in care but not on ART, and 48 (29.6%) were in care and on ART at the time of the index AHI case diagnosis [first bar of Fig 2 and S1 Table]. Care and treatment status was left undetermined after DIS investigation for the remaining 37 (22.8%) previously-diagnosed partners.

**Fig 3:**
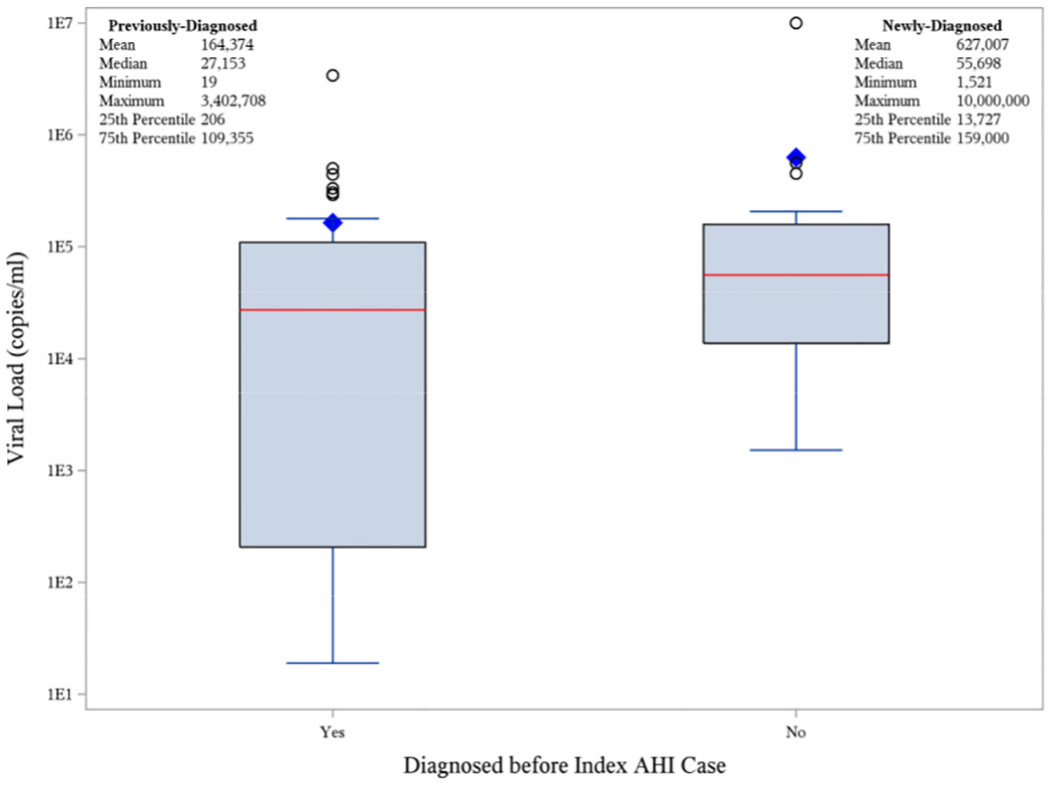
HIV-infected partner viral load (VL) at the time of the Index AHI case diagnosis by diagnosis status. The closest VL within 6 months before (previously-diagnosed partners only) to 2 months after the index AHI diagnosis date was collected for HIV-infected partners and dichotomized by status-aware versus status-unaware partners.

Overall, 40 (24.7%) previously-diagnosed partners had a VL reported in a NC surveillance database 6 months before to 2 months after the index AHI case diagnosis; 19 (33.9%) newly-diagnosed partners (4 AHI and 15 CHI) had a VL in the 2 months after the index AHI case diagnosis. An additional 31 (19.1%) previously-diagnosed partners had a VL reported more than 6 months before the index AHI case diagnosis, suggesting potential loss from care. The partners’ median VL near the time of the index AHI case diagnosis was higher in newly-diagnosed (55,698 copies/ml [range 1,521–10,000,000]) as compared to previously-diagnosed partners (27,153 copies/ml [range 19–3,402,708]) [Fig 3]. Among the 40 previously-diagnosed partners in care and with an available VL, 30 (75.0%) were unsuppressed in the period 6 months before to 2 months after the index AHI case diagnosis, 5 (16.7%) of whom were verified as being infected with HIV within 1 year from the index AHI case. Of the 10 virally suppressed partners at the time of their most recent VL, 4 had detectable VLs in the year surrounding the index AHI case diagnosis, indicating unsustained suppression. None of these partners were diagnosed with recent HIV infection.

#### Likely Transmitting Partner Estimates

A total of 106 index AHI cases named only 1 potential HIV-infected transmitting partner with all other partners testing negative (Type A), 68 named >1 potential transmitting partner with at least one confirmed HIV-infected partner (Type B), and 127 named only potential transmitting partners of unknown status (Type C). The remaining index AHI cases either did not name any partners during the 8 weeks prior to their diagnosis (N = 38) or only named HIV-uninfected partners (N = 19) (Type D) [Table 1].

Among index AHI cases naming only one HIV-infected partner with all other partners testing negative (Type A), over three-quarters of transmission events appeared attributable to contact with previously-diagnosed partners (77.4%, 95% confidence interval (CI) 69.4–85.3%) [2^nd^ bar of Fig 2 and S1 Table]. Among previously-diagnosed partners (N = 82), the proportion of partners reportedly not in care (23.2%, 95% CI 14.0–32.3%), in care and not on ART (26.8%, 95% CI 17.2–36.4%), in care and on ART (23.2%, 95% CI 14.0–32.3%), and with unclassified care and treatment status (26.8%, 95% CI 17.2–36.4%) were roughly equivalent. Of the 38 previously-diagnosed partners with a VL near the time of the index AHI case diagnosis, 94.7% were unsuppressed (N = 36, 95% CI 87.6–100.0%).

Repeated sampling methods resulted in similar diagnosis, care, and treatment status estimates among index AHI cases naming >1 potential transmitting partner with at least one confirmed HIV-infected partner (Type B) [3^rd^ bar of Fig 2 and S1 Table] and when combining these two groups (Types A and B) [4th bar of Fig 2 and S1 Table].

### CHAVI-001

Overall, 55.5% (N = 117) of all index AHI cases identified by the STAT program between May 2006 and December 2011 enrolled in CHAVI-001 and were considered in the complementary analysis [Table 2]. As observed by the STAT program, most index AHI cases were male (87.2%), Black (65.0%) and young (median age = 25 years, IQR 21–36).

Index AHI cases reported 367 partners as a part of the CHAVI-001 investigation (per-index median = 3; range 0–14), of whom 119 (32.4%) were HIV-infected. Compared to STAT investigations, a smaller proportion of HIV-infected partners identified via the CHAVI-001 study were previously-diagnosed (82 (68.9%) previously-diagnosed, 26 (21.8%) new CHI, and 11 (9.2%) new AHI) [Fig 1b]. An additional 126 (34.3%) partners were HIV-uninfected. The status of 122 (33.2%) partners remained unknown (78 anonymous, 44 unlocated or refused testing). Demographics of partners reported during the CHAVI-001 investigation were similar to those observed by the STAT program [Table 2]. Seventy (59.8%) index AHI cases named ≥1 HIV-infected partner; only 47 index AHI cases had ≥1 HIV-infected partner enroll on study and provide samples for phylogenetic analysis. The transmitting partner for 33 (70.2%) of these index AHI cases was phylogenetically verified. One additional phylogenetic linkage was identified between 2 index AHI cases diagnosed 2 years apart. Because neither index AHI case named the other during the CHAVI-001 investigation, we could not rule out a shared or intermediary transmitting partner and excluded this pair from further analysis.

Most of the 33 partners phylogenetically-linked to index AHI cases in CHAVI-001 had been previously-diagnosed (60.6%, 95% CI 43.9–77.3%) [5^th^ bar in Fig 2 and S1 Table], although the contribution is smaller than observed in STAT data. Three linkages were attributable to AHI-to-AHI transmission (9.1%, 95% CI 0.0%-18.9%). All phylogenetically-linked partners were unsuppressed. The median VL of linked partners at transmission was 123,928 copies/ml (range 123,928–2,346,147) for new AHI partners, 62,493 copies/ml (range 2,771–148,042) for new CHI partners, and 72,084 copies/ml (range 10,957–507,795) for previously-diagnosed partners. Although two phylogenetically-linked partners with recognized infection reported a history of ART, neither were on treatment at the time of the index AHI case diagnosis. One partner stopped treatment within 1 month of contact with the index AHI case.

## Discussion

Most observed transmission events in North Carolina appear attributable to contact with previously-diagnosed partners. Roughly one-quarter of these previously-diagnosed partners were reported to be in care and on ART in the 6 months before the index AHI case diagnosis. Not surprisingly, among transmission events that were seemingly attributable to previously diagnosed partners, most (94.7%) were estimated to be from partners confirmed to be virally unsuppressed.

These transmission events occur because many previously-diagnosed persons continue to engage in high-risk behaviors with uninfected persons, as seen in our analyses of the transmission landscape incorporating all identified partners. Over one-third of these identifiable partners were HIV-infected; most were aware of their status prior to the index AHI case diagnosis. Consistent condom use was reported with only 16.7% of previously-diagnosed partners (data not shown). Moreover, previously-diagnosed partners were uncommonly on treatment at the time of contact with the index AHI case, increasing the likelihood of onward transmission [32, 33]. In line with observed HIV infection trends in the South, [34–37] these active transmission networks, as represented by index AHI cases and their partners, were disproportionately populated by young, Black MSM. Engaging this population in care and ensuring receipt of ART is certain to lessen the disease burden in these high-prevalence settings.

The relative contribution of previously undiagnosed partners to onward transmission in this study was lower than estimated in other settings. Model-based estimates predicted 30–75% of all new HIV infections are due to partners unaware of their infection [6–9], a range that is higher than the 22%-38% estimates we obtained in this study.

Our empirical work is limited by a distinct set of biases related to difficult-to-verify assumptions and missing information, as compared to modeling studies. Perhaps most importantly, we had phylogenetic analyses on only a small proportion of the population. The transmission source cannot be verified in partnerships where phylogenetic testing was not done, even if the status of all partners was known and only one was HIV-infected. Furthermore, approximately half of the index AHI cohort named at least one status-unknown or anonymous partner, and over one-third named only unknown/anonymous partners. Undoubtedly, some unknown/anonymous partners transmitted HIV. Status-unknown partners have no record of a positive test in NC surveillance databases under the name provided by the index AHI case and either refused HIV testing or could not be located. HIV infection may be more prevalent among persons refusing HIV testing [38]. If HIV-infected, status-unknown partners are undiagnosed, misnamed by the index AHI case, or were diagnosed outside NC. Anonymous partners did not have any identifiable information, and may be more likely to engage in riskier behaviors and less likely to test for HIV or enter care than identifiable partners.

In this analysis, we presented results (separately) from the STAT program and the CHAVI-001 study to allow for alternate views (with their attendant limitations) on the same questions. While direct comparison across partner care and treatment status was not possible because this information was not collected as part of the CHAVI-001 study, the diagnosis status, specifically the proportion of previously-diagnosed partners, was similar between the random sampling methods used for the STAT data and phylogenetic analyses conducted within the CHAVI-001 study.

Partner services for index AHI cases identified by the STAT program occur close to the transmission event, decreasing the likelihood of recall bias and increasing the accuracy in naming potential transmitting partners and assessing their diagnosis, care, and treatment status. However, DIS assign and report continuum stages of previously-diagnosed partners based on their investigations for the STAT program, preventing any sensitivity analyses surrounding the timing and length of partner care and treatment status. Furthermore, diagnosis during AHI is relatively uncommon in NC, [39] making it difficult to assess any heterogeneity in partner continuum stages that might have existed over the 11 years under analysis.

The contribution of AHI to ongoing transmission is not easily assessed with these data. The duration of AHI is short and partners transmitting during this stage may not be tested until they reach CHI. Approximately one-third of newly-diagnosed partners with available STARHS testing data were classified as having recent infection, suggesting these partners were unaware of their HIV status for a short period of time, with some potentially representing AHI-to-AHI transmission. When both the index case and the partner are diagnosed with AHI, the direction of transmission is difficult to determine, even if phylogenetically linked, [31] leaving open the possibility that the index AHI case transmitted disease to at least some newly-diagnosed partners unaware of their HIV status.

Partner viral suppression status at the time of transmission is difficult to assign. Detectable VL reporting has been mandated by the NC DPH since 1991 [40] and undetectable VL reporting since 2013 [41]. However, actual reporting varies from laboratory to laboratory. Since 2006, the NC DPH estimates that 60–70% of all sites were reporting VLs and that most of these sites were voluntarily reporting both detectable and undetectable VLs (personal communication with Erika Samoff, NC DPH). In our study, only half of the previously-diagnosed partners had an available VL in surveillance databases near the time of the index AHI case diagnosis and approximately 20% of these partners were virally suppressed. The relatively low levels of viral suppression observed among previously-diagnosed partners may be a result of more restrictive treatment guidelines in place prior to 2009 [42]. If partners are truly virally suppressed, HIV transmission is known to be unlikely [1, 43]. Sexually transmitted co-infection, treatment non-adherence or resistance provide possible explanations for potential rebound in virus in the period between the last suppressed VL and contact with the index case [44–46].

Engagement (and re-engagement) in care and early initiation of treatment should remain a high priority to prevent HIV transmission. In both surveillance data from the STAT program and phylogenetic data collected via the CHAVI-001 study, a substantial proportion of HIV transmission appears attributable to contact with previously-diagnosed partners. Interventions to find previously-diagnosed persons not in care and facilitate receipt of consistent care and immediate treatment should have a tremendous impact on improving health and reducing HIV incidence in this and similar settings.

## Supporting Information

S1 Table HIV care continuum stages of HIV-infected partners. (DOCX)
