# Supplementary material for: Ongoing HIV Transmission and the HIV Care Continuum in North Carolina
Source: PLoS One. 2015 Jun 4;10(6):e0127950. doi: 10.1371/journal.pone.0127950 (PMC4456412; doi:10.1371/journal.pone.0127950)
Supplement: S1 Table — (DOCX) [file pone.0127950.s001.docx]

**S1 Table. HIV care continuum stages of HIV-infected partners**

|  | **Transmission Landscape** | | | **Most Likely Transmission Source:**  **Unconfirmed Linkage** | | | | | | | | | | **Most Likely Transmission Source:**  **Confirmed Linkage** | | |
| --- | --- | --- | --- | --- | --- | --- | --- | --- | --- | --- | --- | --- | --- | --- | --- | --- |
|  | STAT:  All Identified HIV+ Partners | | | STAT Type A:  1 potential transmitting partner with all other partners testing HIV-negative | | | STAT Type B:  >1 potential transmitting partner with at least 1 confirmed HIV-infected partner | | | STAT Types A+B:  ≥1 potential transmitting partner with at least 1 confirmed HIV+ partner | | | CHAVI-001:  Phylogenetically-Linked Partner | | | |
|  | HIV+ Partner N=218 | | | Index AHI N=106 HIV+ Partner N=106 | | | Index AHI N=68 HIV+ Partner N=112 | | | Index AHI N=174  HIV+ Partner N=218 | | | Index AHI N=33 HIV+ Partner N=33 | | | |
|  | N | $\hat{p}$ | 95% CI | N | $\hat{p}$ | 95% CI | N | $\hat{p}$ | 5th & 95th percentiles | N | $\hat{p}$ | 5th & 95th percentiles | N | | $\hat{p}$ | 95% CI |
| New AHI | 11 | 0.050 | 0.021-0.080 | 1 | 0.001 | 0.000-0.030 | 10 | 0.076 | 0.059-0.103 | 11 | 0.036 | 0.029-0.046 | 3 | | 0.091 | 0.000-0.189 |
| New CHI | 45 | 0.206 | 0.153-0.260 | 23 | 0.217 | 0.139-0.295 | 22 | 0.163 | 0.118-0.206 | 45 | 0.196 | 0.178-0.213 | 10 | | 0.303 | 0.146-0.460 |
| Previously-diagnosed, not in care | 26 | 0.119 | 0.076-0.162 | 19 | 0.179 | 0.106-0.252 | 7 | 0.078 | 0.059-0.103 | 26 | 0.140 | 0.132-0.149 | -- | | -- | -- |
| Previously-diagnosed in care, not on ART | 51 | 0.234 | 0.178-0.290 | 22 | 0.208 | 0.130-0.285 | 29 | 0.278 | 0.235-0.309 | 51 | 0.235 | 0.218-0.247 | -- | | -- | -- |
| Previously-diagnosed, in care, on ART | 48 | 0.220 | 0.165-0.275 | 19 | 0.179 | 0.106-0.252 | 29 | 0.244 | 0.206-0.279 | 48 | 0.204 | 0.190-0.218 | -- | | -- | -- |
| *Previously-diagnosed Unclassified Care & Treatment* | *37* | *0.170* | *0.120-0.220* | *22* | *0.208* | *0.130-0.285* | *15* | *0.162* | *0.132-0.191* | *37* | *0.190* | *0.178-0.201* | *20* | | *0.606* | *0.439-0.773* |
